# *γ/δ* Cells in Fetal, Neonatal, and Adult Rat Lymphoid Organs

**DOI:** 10.1155/1995/73127

**Published:** 1995

**Authors:** P. Kühnlein, A. Vicente, A. Varas, T. Hünig, A. Zapata

**Affiliations:** 1 lnstitut für Virologie und Immunbiologie der Universität Würzburg Versbacher Str. 7 Würzburg D-97078 Germany; 2 Department of Cell Biology Faculty of Biology Complutense University Madrid 28040 Spain

**Keywords:** *γδ* T cells, rat, ontogeny

## Abstract

In the present study, we have analyzed the appearance and maturation of  *γ/δ* T cells, recognized with a new mAb V65, in the central and peripheral lymphoid organs of fetal, neonatal, and adult Wistar rats. Cytofluorometrical analysis demonstrated the first V65^+^ *γ/δ* T cells in the thymus of 16-17-day embryonic rats, although by immunohistology, they were identified only in 19-day rat embryos in both the cortico-medullary border and thymic medulla. Phenotypically, *γ/δ* thymocytes from fetal and neonatal thymus expressed CD3, CD2, and CD5, but only 60-80% were CD8^+^ and approximately 40-50% expressed the *α* chain (p55) of the IL-2R. In the periphery, the immunohistological study identified for the first time ,*γ/δ* T cells in the splenic white pulp and the gut of 21-day fetal rats, where they occurred within the epithelium as well as in the lamina propria. After birth, *γ/δ* lymphocytes appeared in the skin, where they were present as dendritic epidermal T cells in increasing numbers during postnatal life. Whereas these *γ/δ* T cells formed the predominant T-cell population in the rat skin, *γ/δ* T cells in peripheral lymphoid organs, BALT, or the gut only represented a minor T-cell population. These results are discussed in comparison to *γ/δ* T cells of other vertebrate species.


*Developmental Immunology*, 1995, Vol 4, pp. 181–188
Reprints available directly from the publisher
Photocopying permitted by license only

© 1995 Harwood Academic Publishers GmbH
Printed in Singapore

### γ/δ T Cells in Fetal, Neonatal, and Adult Rat Lymphoid Organs

P. KÜHNLEIN,[†] A. VICENTE,[‡] A. VARAS,[‡] T. HÜNIG,[†] and A. ZAPATA*[‡]

[†]*Institut für Virologie und Immunbiologie der Universität Würzburg, Versbacher Str. 7, D-97078 Würzburg*
[‡]*Department of Cell Biology, Faculty of Biology, Complutense University, 28040 Madrid, Spain*

In the present study, we have analyzed the appearance and maturation of γ/δ T cells, recognized with a new mAb V65, in the central and peripheral lymphoid organs of fetal, neonatal, and adult Wistar rats. Cytofluorometrical analysis demonstrated the first V65$^+$ γ/δ T cells in the thymus of 16–17-day embryonic rats, although by immunohistology, they were identified only in 19-day rat embryos in both the cortico-medullary border and thymic medulla. Phenotypically, γ/δ thymocytes from fetal and neonatal thymus expressed CD3, CD2, and CD5, but only 60–80% were CD8$^+$ and approximately 40–50% expressed the α chain (p55) of the IL-2R. In the periphery, the immunohistological study identified for the first time γ/δ T cells in the splenic white pulp and the gut of 21-day fetal rats, where they occurred within the epithelium as well as in the lamina propria. After birth, γ/δ lymphocytes appeared in the skin, where they were present as dendritic epidermal T cells in increasing numbers during postnatal life. Whereas these γ/δ T cells formed the predominant T-cell population in the rat skin, γ/δ T cells in peripheral lymphoid organs, BALT, or the gut only represented a minor T-cell population. These results are discussed in comparison to γ/δ T cells of other vertebrate species.

KEYWORDS: γδ T cells, rat, ontogeny.

### INTRODUCTION

Two separate T-cell lineages, called α/β and γ/δ T cells, have been identified based on the type of heterodimeric antigen receptors expressed (reviewed by Haas et al., 1993). In all vertebrates studied so far including humans (Lew et al., 1986; Nakanishi et al., 1987), mice (Bluestone et al., 1987), chickens (Bucy et al., 1988; Sowder et al., 1988), rats (Lawetzky et al., 1990; Vaage et al., 1990; Kühnlein et al., 1994), sheep (Hein and Mackay, 1991), cattle (Mackay and Hein, 1989), and pigs (Hirt et al., 1990; Saalmüller et al., 1990), the existence of both α/β and γ/δ T cells has been validated. In rats, γ/δ T cells have so far only been indirectly identified as CD3$^+$ α/β TCR$^-$ cells (Lawetzky et al., 1990; Vaage et al., 1990). Whereas this approach is feasible for flow cytometric analysis, it has only limited applications in immunohistological studies. Recently, a monoclonal antibody to a constant determinant of the rat γ/δ TCR has been isolated (Kühnlein et al., 1994). In the present study, we have used this reagent to analyze by immunohistology the appearance and distribution of γ/δ T cells in both central and peripheral lymphoid organs of fetal, neonatal, and adult Wistar rats. In addition, the appearance of γ/δ T cells during fetal development of the rat thymus was evaluated by flow cytometry.

* Corresponding author.

### RESULTS

### Immunohistology

*Thymus*

The first γ/δ T cells were observed in day-19 fetal rat thymus. As shown for a day-21 fetal thymus in Fig. 1, they occurred mainly along the cortico-medullary border and in the medulla. These fetal γ/δ thymocytes frequently occurred in association with blood vessel walls (Fig. 2). In addition, some V65-positive cells appeared occasionally in the connective tissue trabeculae (not shown).

In both postnatal and adult rats, the γ/δ T cells appeared scattered throughout the thymic parenchyma although they were more abundant in both the cortico-medullary border and the medulla (Fig. 3). In the cortex, as mentioned before for fetal medullary γ/δ T cells, they were frequently found associated with blood vessels (Fig. 4), and, with very low frequency, also in the subcapsular area (Fig. 5). Although the number of V65-positive cells appeared to increase gradually throughout the first weeks of thymus development, γ/δ T cells always represent a very small minority of thymocytes.

### *Peripheral Lymphoid Organs*

In the spleen, the first γ/δ T cells appeared on day 21 of fetal life closely associated with the large blood vessels around which the periarteriolar-associated lymphoid sheaths (PALS) will be organized after birth (Fig. 6). Their location in the PALS became apparent after birth (Fig. 7), already resembling the adult condition.

As in the spleen, the first γ/δ T cells occurred in the fetal gut from embryonic day 21. In that stage, they occupied mainly the lamina propria (Fig. 8), but after birth, the V65-positive cells appeared also as intraepithelial elements throughout the gut (Fig. 9). Although during the first 2 weeks of postnatal life, their number increased considerably (Fig. 10), it never reached that of α/β T lymphocytes (not shown) in either the intestinal epithelium or the lamina propria. A few γ/δ T cells were also found in the T-dependent areas of Peyer's patches (not shown).

In the skin of Wistar rats, γ/δ T cells became detectable only after birth (Fig. 11). These cells, which were always located in the epidermis, gradually increased in number and staining intensity until the adult condition was reached (Fig. 12), in which frequently they formed cell clusters (Fig. 13). The skin was the only organ studied in which the number of γ/δ T cells exceeded that of α/β T lymphocytes (not shown).

An immunohistological survey of γ/δ T cells in mesenteric and cervical lymph nodes from postnatal rats showed that in both cases, only a few γ/δ T lymphocytes were scattered randomly in the T-dependent areas, mainly in the so-called deep cortex (Fig. 14). From the second week of postnatal life, their number increased considerably.

In the lung of Wistar rats, a few γ/δ T cells occurred in the bronchus-associated lymphoid tissue (BALT), but not in the pulmonary parenchyma (Fig. 15). In contrast to α/β T cells that are found throughout the BALT (not shown), γ/δ T lymphocytes occurred mainly in the periphery of lymphoid aggregates from day 15 of postnatal life (Fig. 15).

### FACS Analysis

By immunofluorescence and flow cytometry, the first γ/δ cells were detected in the thymus of 16–17-day embryonic rats. At this time point, they represented 1.3% of total thymocytes (Fig. 16a). During the following days, no significant differences in frequency were observed (Figs. 16b, 16c), although the absolute number of $V65^+$ cells increased along with the total cellularity of the thymus.

All γ/δ thymocytes also expressed CD3 (Figs. 16a, 16b, 16c) CD2 (Fig. 16d), and CD5 (Fig. 16e), but only 60–80% of them were $CD8^+$ ($OX8^+$ cells) (Fig. 16f). The remaining γ/δ thymocytes (about 25%) were double-negative ($CD4^-CD8^-$) cells (Figs. 16f, 16g). In all stages studied, 40–50% of γ/δ thymocytes expressed the α chain of the IL-2 receptor (Fig. 16h).

### DISCUSSION

The current study is the first direct description of γ/δ T cell development in rats. As assessed by immunohistology, the first γ/δ T cells appeared during ontogeny in the thymus, and by flow cytometry, they can be detected as early as day 16 in this organ. The delayed appearance of γ/δ cells in the periphery (day 21 in fetal spleen and gut, and postnatally in skin, and even later in BALT) confirms and extends earlier results on $CD3^+$ TCR $\alpha/\beta^-$ cells (Lawetzky et al., 1990). This finding does not necessarily mean that all rat γ/δ T cells are thymus-derived. Thus, phenotypic analysis of fetal day 21 (and adult, not shown) thymocytes revealed a uniform expression of CD2 and CD5, and expression of CD8 on the majority of the cells. This phenotype corresponds to that of rat γ/δ T cells isolated from peripheral lymphoid organs, but is distinct from that of intestinal epithelial γ/δ T cells that may represent a distinct lineage (Kühnlein et al., 1994). The predominance of $CD4^-8^+$ γ/δ thymocytes in rats confirms earlier data by Gottlieb et al. (1991).

form a clustered network presumably after undergoing substantial proliferation *in situ*. Payer et al. (1991) emphasize that this proliferation is needed to explain the establishment of the mature population of dendritic epidermal γ/δ T cells in postnatal skin from a small number of precursors from fetal thymus (Payer et al., 1991). With regard to rat DETC development, those results need, however, further validation because an adequate morphometrical study has not been realized and we did not observe mitotic figures in the epidermal V65$^+$ cells of rat skin.

In contrast to findings in mice where up to 50% of intraepithelial lymphocytes are γ/δ T cells (Guy-Grand and Vassalli, 1993), and chicken, where they represent the major intraepithelial lymphocyte (IEL) population (Bucy et al., 1988; Gómez del Moral, 1994), γ/δ lymphocytes are not the predominant T-cell subpopulation in the gut epithelium of rats. As recently shown by flow cytometry (Kühnlein et al., 1994), we found that they rather represent a minor cell population (5–10%), similar to the situation in humans (Spencer et al., 1989). Because this comes close to the frequency of γ/δ T cells in other peripheral lymphoid tissues (Kühnlein et al., 1994), it is not surprising that the profound preponderance of γ/δ T cells observed in mouse gut epithelium as compared to the lamina propria was not evident in

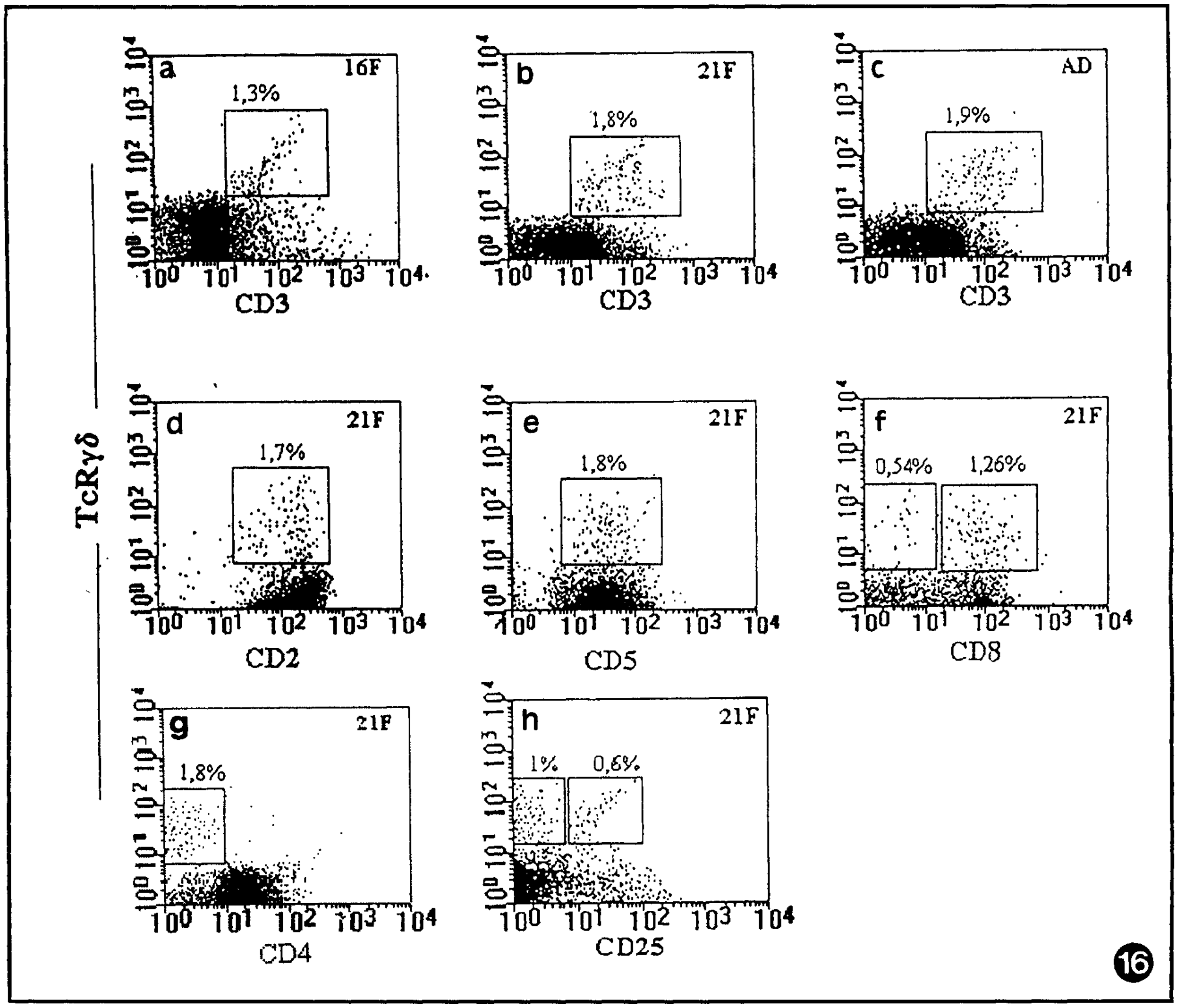

FIGURE 16. Expression of various T-cell markers on rat γδ T-cells: (a) CD3 (16-day fetal rat); (b) CD3 (21F); (c) CD3 (adult); (d) CD2 (21F); (e) CD5 (21F); (f) CD8 (21F); (g) CD4 (21F); and (h) CD25 (21F).

the rat. A similar location of γ/δ and α/β T cells was also observed in the rat spleen, where they occupy the periarteriolar-associated lymphoid sheaths. In contrast, in the spleen of both humans (Bucy et al., 1989) and chickens (Bucy et al., 1988), γ/δ T lymphocytes predominate in the red pulp and α/β T cells occupy mainly the PALS. It is unknown if these differences in distribution reflect distinct functional capacities of γ/δ T cells.

In summary, our immunohistochemical results demonstrate remarkable differences in the distribution of γ/δ T lymphocytes in the periphery of rats as compared to other species so far investigated. The relevance of the species-specific distribution of at least some γ/δ T cells subsets will only be understood once the functional role of these cells has been clarified.

### MATERIALS AND METHODS

### Animals

Wistar rats were bred in our own facilities. Male and female rats were mated overnight and females were examined the next morning. The detection of a vaginal plug was designated as day 0 of gestation.

### Antibodies

The generation and characterization of mAb V65 to a constant determinant of the rat γ/δ TCR has been described in detail (Kühnlein et al., 1994).

### Immunohistochemical Techniques

Lymphoid organs, including thymus, spleen, mesenteric, and cervical lymph nodes, gut, Peyer's patches, lung, and skin, were removed from embryonic, neonatal, and adult Wistar rats and frozen in liquid nitrogen. Five-micrometer cryosections of these organs were fixed for 10 min in acetone and then incubated for 1 h with mAb V65. Endogenous peroxidase was blocked with 1% $H_2O_2$ in methanol for 15 min. After washing in PBS, the histological sections were incubated with a 1:40 solution of peroxidase-conjugated rabbit anti-mouse Ig in PBS (Dakopatts, Glostrup, Denmark) supplemented with 1:100 normal rat serum in PBS. After washing, the peroxidase reaction was developed with 0.05% 3,3′ diaminobenzidine (Sigma, St. Louis, MO) in PBS with 0.1% $H_2O_2$ for 10 min. The sections were counterstained with methylene blue.

### Thymic-Cell Suspensions

Thymuses were dissected from fetal and neonatal rats using a stereoscopic microscope. Thymocyte suspensions were prepared in cold PBS 4% FCS by passing either thymic fragments through a stainless steel screen or by passing fetal thymic lobes through hypodermic needles of decreasing size. In both cases, cell viability was ≥98%. Six to 10 fetal thymuses from a pregnant rat were pooled for each analysis.

### Immunofluorescence and Flow Cytometry

For two-color staining, $3 \times 10^5$ cells were successively incubated for 20 min in 50-μl unconjugated mAb V65 at a saturating concentration, normal rabbit serum (1:100 PBS) to avoid nonspecific binding, phycoerythrin- (PE-) conjugated rabbit $F(ab')_2$ anti-mouse IgG (RAM-PE) (Southern Biotechnology, Birmingham, AL), normal mouse serum (1:100 PBS), and the following FITC-conjugated mAbs: OX8 (CD8); OX38 (CD4); OX34 (CD2); OX19 (CD5); G4-18 (CD3); and OX39 (CD25) (Pharmingen, Oxon, UK). All staining steps were performed at 0–4°C in PBS-2% FCS. After washing twice and treatment with propidium iodide to label dead cells, samples were analyzed on a FACScan Flow Cytometer (Becton Dickinson, Mountain View, CA). Background fluorescence was determined using an irrelevant antibody.

*(Received November 22, 1994)*

*(Accepted January 13, 1995)*

### REFERENCES

Asarnow D.M., Kuziel W.A., Bonyhadi M., Tigeelar R.E., Tucker P.W., and Allison J.P. (1988). Limited diversity of γδ antigen receptor genes of Thy $1^+$ dendritic epidermal cells. Cell **55:** 837–847.

Bluestone J.A., Pardoll D., Sharrow S.O., and Fowlkes B.J. (1987). Characterization of murine thymocytes with CD3-associated T-cell receptor structures. Nature **326:** 82–84.

Bucy R.P., Chen C.H., Cihak J., Lösch U., and Cooper M. (1988). Avian T cells expressing γδ receptors localize in the splenic sinusoids and the intestinal epithelium. J. Immunol. **141:** 2200–2205.

Bucy R.P., Chen C.H., and Cooper M. (1989). Tissue localization and CD8 accessory molecule expression of T $\gamma\delta$ cells in humans. J. Immunol. **142:** 3045–3049.

Campana D., Janossy G., Coustain-Smith E., Amlot P.L., Tian W.T., Ip S., and Wong L. (1989). The expression of T cell receptor-associated proteins during T cell ontogeny in man. J. Immunol. **142:** 57–66.

Chen C.H., Cihak J., Lösch U., and Cooper M. (1988). Differential expression of two cell receptors, TcR1 and TcR2, on chicken lymphocytes. Eur. J. Immunol. **18:** 539–543.

Elbe A., Tschachler E., Steiner G., Binder A., Wolff K. and Stingly G. (1989). Maturation steps of bone marrow-derived dendritic murine epidermal cells. Phenotypic and functional studies on Langerhans cells and Thy-$1^+$ dendritic epidermal cells in the perinatal period. J. Immunol. **143,** 2431–2438.

Farr A., Hosier S., Nelson A., Itohara S., and Tonegawa S. (1990). Distribution of thymocytes expression $\gamma\delta$ receptors in the murine thymus during development. J. Immunol. **144:** 492–498.

Gómez del Moral (1994). Ontogenia de la tonsila cecal de pollo. Análisis citofluorimétrico e inmunohistológico. Masters thesis, Complutense University, Spain.

Gottlieb W.H., Takacs L., Finch L.R., Kopp W., Weissman A.M., and Durum S.K. (1991). CD8 $\gamma\delta$ cells: Presence in the adult rat thymus and generation in vitro from CD4-CD8-thymocytes in the presence of interleukin 2. Cytokine **3:** 598–608.

Guy-Grand D., and Vassalli P. (1993). Gut intraepithelial T lymphocytes. Curr. Op. Immunol. **5:** 247–252.

Haas W., Pereira P., and Tonewaga S. (1993). Gamma/delta cells. Annu. Rev. Immunol. **11:** 637–685.

Havran W.L., and Allison J.P. (1988). Developmentally ordered appearance of thymocytes expressing different T-cell antigen receptors. Nature **335:** 443–445.

Havran W.L., and Allison J.P. (1990). Origin of Thy-1 + dendritic epidermal cells of adult mice from fetal thymic precursors. Nature **344:** 68–70.

Hein W.R. and Mackay C.R. (1991). Prominance of $\gamma\delta$ T cells in the ruminant immune system. Immunol. Today **12:** 30–34.

Hirt W., Saamuller A., and Reddehase M.J. (1990). Distinct $\gamma\delta$ T cell receptors define two subsets of circulating porcine $CD2^-CD4^-CD8^-$ T lymphocytes. Eur. J. Immunol. **20:** 265–269.

Itohara S., Nakawishi N., Kanagawa O., Kubo R., and Tonewaga S. (1989). Monoclonal antibodies specific to native murine T-cell receptor $\gamma\delta$: Analysis of $\gamma\delta$ T cells during ontogeny and in the peripheral lymphoid organs. Proc. Natl. Acad. Sci. USA **86:** 5094–5098.

Kühnlein P., Park J.-H., Herrmann T., Elbe A., and Hünig T. (1994). Identification and characterization of rat $\gamma/\delta$ T lymphocytes in peripheral lymphoid organs, small intestine, and skin with a monoclonal antibody to a constant determinant of the $\gamma/\delta$ T cell receptor. J. Immunol. **153:** 979–986.

Lawetzky A., Tiefenthaler G., Kubo R., and Hünig T. (1990). Identification and characterization of rat T cell subpopulations expressing T cell receptors $\alpha\beta$ and $\gamma\delta$ Eur. J. Immunol. **20:** 343–349.

Leclercq G., De Smedt M., and Plum J. (1992). Cytokine production and responsiveness of fetal T-cell receptor V$\gamma$3 thymocytes. Scand. J. Immunol. **36:** 833–841.

Lew A.M., Pardoll D.M., Maloy W.L., Fowlkes B.J., Kruisbeek A., Chen S.-F., Germain R.N., Buestone J.A., Schwartz R.H., and Coligan J.E. (1986). Characterization of T cell receptor gamma chain expression in a subset of murine thymocytes. Science **234:** 1401–1405.

Mackay C.R., and Hein W.R. (1989). A large proportion of bovine T cells express the $\gamma\delta$ T cell receptor and show a distinct tissue distribution and surface phenotype. Int. Immunol. **1:** 540–545.

Nakanishi N., Maeda K., Ito K., Heller M., and Tonewaga S. (1987). T$\gamma$ protein is expressed on murine fetal thymocytes as a disulfide-linked heterodimer. Nature **325:** 720–723.

Payer E., Elbe A., and Stingl G. (1991). Circulating CD3 + /T cell receptor V$\gamma$3 + fetal murine thymocytes home to the skin and rise to proliferating dendritic epidermal T cells. J. Immunol. **146:** 2536–2543.

Romani N., Schuler G., and Fritsch P. (1986). Ontogeny of Ia-positive and Thy-1 positive leukocytes of murine epidermis. J. Invest. Dermatol. **86:** 129–135.

Saamuller A., Hirt W., and Reddehase M.J. (1990). Porcine $\gamma\delta$ T lymphocytes subsets differing in their propensity to home to lymphoid tissue. Eur. J. Immunol. **20:** 2343–2346.

Sato K., Ohtsuka K., Watanabe H., Asakura H., and Abo T. (1993). Detailed characterization of $\gamma\delta$ T cells within the organs in mice: classification into three groups. Immunology **80:** 380–387.

Schimpl A., Hünig T., Elbe A., Berberich I., Krämer S., Merz H., Feller A.C., Sadlack B., Schorle H., and Horak I. (1994). Development and function of the immune system in mice with targeted disruption of the interleukin-2 gene. *In:* Transgenesis and Immunodeficiency, Blüthmann H., Ed. (New York: Academic Press), pp. 191–202.

Sowder J.T., Chen C.H., Lanier C., Ager L., Chan M.M., and Cooper M. (1988). A large subpopulation of avian T cells express a homologue of the mammalian T $\gamma\delta$ receptor. J. Exp. Med. **167:** 315–322.

Spencer J., Isaacson P.G., Diss T., and MacDonald T. (1989). Expression of disulfide-linked and non-disulfide-linked forms of the T cell receptor $\gamma\delta$ heterodimer in human intestinal intraepithelial lymphocytes. Eur. J. Immunol. **19:** 1335–1339.

Tanaka T., Takeuchi Y., and Shiohara T. (1992). *In utero* treatment with monoclonal antibody to IL-2 receptor $\beta$-chain completely abrogates development of Thy-$1^+$ dendritic epidermal cells. Int. Immunol. **4:** 487–491.

Vaage J.T., Dissen E., Ager A., Roberts I., Fossum S., and Rolstad B. (1990). T cell receptor-bearing cells among rat intestinal intraepithelial lymphocytes are mainly $\alpha\beta^+$ and are thymus dependent. Eur. J. Immunol. **20:** 1193–1196.

